# Supplementary material for: Evolutionarily recent retrotransposons contribute to schizophrenia
Source: Transl Psychiatry. 2023 May 27;13:181. doi: 10.1038/s41398-023-02472-9 (PMC10224989; doi:10.1038/s41398-023-02472-9)
Supplement: Supplementary file 1 — Supplementary Material [file 41398_2023_2472_MOESM1_ESM.docx]

**Evolutionarily recent retrotransposons contribute to schizophrenia**

Giorgia Modenini, Paolo Abondio, Guia Guffanti, Alessio Boattini, Fabio Macciardi

**SUPPLEMENTARY MATERIALS AND METHODS**

**Dorso-Lateral Pre-Frontal Cortex (DLPFC) samples**

DNA from the DLPFC of ten schizophrenic patients (SCZ) and ten psychiatrically healthy controls (CTRL) has been obtained from the UCI Brain Bank. Donors or their first-degree relatives signed an informed consent to the UCI Brain Bank to have their tissues donated for scientific research, under an UCI-IRB approved protocol. Our sample includes 14 men and 6 women, whose ages at death ranged from 31 to 68 (average = 46.1 ± 11.4 [of which CTRL: 48 ± 13, SCZ: 44.3 ± 10, p-val = ns]). Cases and controls were matched for sex and age. Brain tissues have been collected within a mean postmortem interval (PMI) of 19 ± 4 hours. All samples presented a pH from 6.0 to 7.1 (average 6.4 ± 0.3). Specimens were checked for the presence of other potential disease states as described in Guffanti et al., (2018). Following dissection, samples were flash frozen. We extracted DNA from 80-100 ng DLPFC frozen samples using the Qiagen DNA kit… DNA concentration was assessed using a NanoDrop specttrophotometer.

**Whole genome sequencing**

The 20 DLPFC DNA samples have been sequenced by Illumina using an HumanOmni2.5-8v1 platform, for generating a mean yield of 131.94 Gb/genome with a mean bases’ percentage >= Q30 of 85.33%. Fasta files were retrieved from the Illumina sequences. AdapterRemoval (Lindgren, 2012) was used to remove adapters from the fastq files. Alignment to the human reference genome hs37d5 ([http://ftp.1000genomes.ebi.ac.uk/vol1/ftp/technical/reference/](http://ftp.1000genomes.ebi.ac.uk/vol1/ftp/technical/reference/phase2_reference_assembly_sequence)) was performed with BWA-mem (Li and Durbin, 2009). After sorting and merging with Samtools (Li et al., 2009), MarkDuplicates (a tool provided within the PicardTools package: <http://broadinstitute.github.io/picard/>) was applied.

We applied the GATK best practices to generate VCF files (https://gatk.broadinstitute.org/hc/en-us/articles/360035894711-About-the-GATK-Best-Practices) that include SNPs as well as Indels.

**Identification of TEs in DLPFC and in the 1000 Genomes Project samples: MELT**

We looked at non-reference Retrotransposon Insertions (RIs: Alu, LINE1 and SVA) with the Mobile Element Locator Tool (MELT) v.2.1.5 (Gardner et al, 2017), using MELT-Split with default parameters on our twenty high-coverage genomes (Supplementary Table 1)

To analyze the geographic variability of the putative schizophrenia-related RIs, we additionally selected 125 samples from the “1000 Genomes Project” phase 3 (The 1000 Genomes Project Consortium, 2015). In particular, we selected 25 samples from each of five populations (Utah Residents with Northern and Western European Ancestry, CEU; Han Chinese in Beijing, CHB; Indian Telugu, ITU; Luhya in Kenya, LWK; Yoruba in Nigeria, YRI) which are representative of worldwide genomic variability. Individuals were selected avoiding relatives and excluding samples with an excessive number of discordant read pairs (DRPs) [see Supplementary Materials in Gardner et al., (2017)]. For the complete list of individuals see Supplementary Table 1.

These 125 samples were analyzed with MELT jointly with SCZ and CTRL samples. Only “PASS” sites were included in a single final VCF file and only RIs mapping in genic or regulatory regions (introns, exons, promoters, terminators and UnTranslated Regions, UTRs) on autosomal chromosomes were considered for further analyses. Fisher tests of independence were performed to identify which RIs revealed significantly different frequencies in SCZ and CTRL. Tests were performed with one and two degrees of freedom, respectively, for allelic and genotype frequencies. RIs that yielded nominally significant tests (pval < 0.05) at least for allele and/or genotype frequencies were considered as putatively related with schizophrenia.

**PCA and Admixture**

In order to assess the genetic relationships among the individuals included in our dataset, as well as their ancestry, we implemented a principal component analysis (PCA) and ADMIXTURE analysis (Alexander, Novembre and Lange, 2009), both on the whole variant dataset (single nucleotide polymorphisms, SNPs, and TEs) and the nrTE-based only. Quality control (QC) was performed with the PLINK software (Purcell et al, 2007), including the removal of genetic elements belonging to the sex chromosomes, a check for the proportion of missing data (using the commands --geno and --mindwith a threshold of 0.01), the respect of Hardy-Weinberg equilibrium after Bonferroni correction for multiple testing (--hwe 0.01/α, where α is equal to the number of variants remaining in the dataset at this stage of the QC procedure), the removal of rare variants (--maf 0.01) and an assessment of linkage disequilibrium along the genome, using a sliding window of 50bp, a moving step of 5bp and a threshold value of 0.1 (--indep-pairwise 50 5 0.1).

After QC, the whole variant dataset is reduced to 164.601 variants, while the nrTE-based dataset (obtained by extracting TEs’ positions from the whole variant dataset) contained 3.211 variants. For both datasets, PCA was performed by applying a series of file format conversions and computations as required by the *convertf* and *smartpca* tools from the EIGENSOFT package v6.0.1 (Price et al, 2006).

Similarly, the ADMIXTURE software (Alexander, Novembre and Lange, 2009) was employed to estimate the shared genetic ancestry across populations. We tested between 2 and 7 putative ancestral components (= K), performing 50 iterations of each run to minimize the estimation error and maximize the log-likelihood of each ancestry estimate.

**Haplotype estimation**

We also performed a haplotype reconstruction procedure on the whole variant dataset to contextualize the genotyped non-reference TEs into their local genetic environment and evaluate the frequency of the corresponding haplotypes within the DLPFC cohort. After checking for missing data and Hardy-Weinberg equilibrium expectations, ambiguous SNPs (carrying an A/T or C/G combination of alleles, for which the maternal and paternal chromosome , as well as, the strand cannot be unequivocally defined) were removed. Information about the ancestral or derived nature of each SNP was deduced by using a reconstructed reference human genome sequence. Briefly, ancestral/derived states of each allele were previously assigned by aligning the human reference sequence hs37d5 (see Supplementary Materials and Methods) with the five available Ensembl Compara primates EPO reference genome sequences (Herrero et al, 2016): bonobo (*Pan paniscus*), chimpanzee (*Pan troglodytes*), gorilla (*Gorilla gorilla*), macaque (*Macaca fascicularis*) and orangutan (*Pongo abelii*). Only alleles conserved in all the compared genomes are considered as ancestral. In this framework, for non-reference TEs, the derived allele corresponds to the presence of the element. Haplotype estimation was finally performed with the SHAPEIT software version 1.9 (Delaneau, Zagury and Marchini, 2013) on a dataset of 8.331.932 variants (SNPs and TEs). Haplotype estimation also includes haplotype phasing.

**Association test with Beagle**

An association test was performed using Beagle v3.3.2 (S.L. Browning and B.L. Browning, 2007) on the TEs with significantly different allelic and/or genotype frequencies between SCZ and CTRL. First, we selected regions of interest using VCFtools (Danececk et al., 2011) and phased those regions with Beagle v5.1 (S.L. Browning and B.L. Browning, 2007; B.L. Browning, Y. Zhou and S.R. Browning, 2018) as described in the manual. Then, we converted the phased VCF file into the Beagle format with the Beagle utility vcf2beagle (https://faculty.washington.edu/browning/beagle_utilities/) and included the case status (“schizophrenic”) in the first row of the .bgl file. Lastly, we performed the association test with Beagle v3.3.2 as described in the manual and then checked for significant results with the cluster2haps option.

**In silico functional inferences on non-reference TEs’ role**

TEs can act as cis regulatory elements by, for example, modifying the expression of nearby genes and inducing alternative splicing. Therefore, we checked if non-reference TEs may act as eQTLs (expressions Quantitative Trait Loci) and/or sQTLs (alternative splicing Quantitative Trait Loci), by comparing our significant results with those from Cao et al. (2020), based on the GTEx dataset (ref).

Moreover, we verified whether the statistically significant non-reference TEs (i.e., nrTEs with significantly different allele/genotype frequencies between cases and controls) are located close to genes previously studied in the context of schizophrenia in available literature.

We also compared our whole dataset of nrTEs with the lists of HARs as originally identified by Pollard et al. (2006a, b), Prabhakar et al. (2006), Bird et al. (2007), Capra et al. (2013) and Gittelman et al. (2015), to check if some of the identified TEs are located in those regions.

**SUPPLEMENTARY FIGURES**

**A**


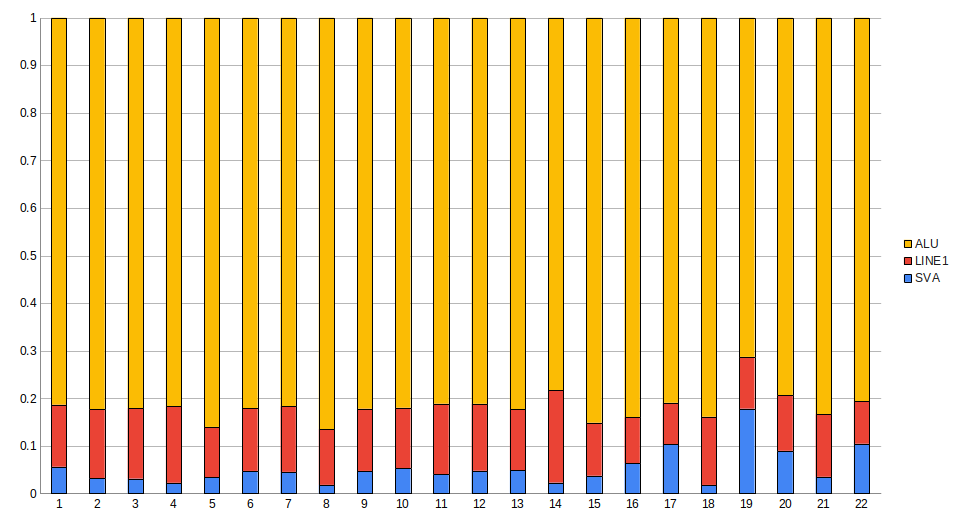


**B**


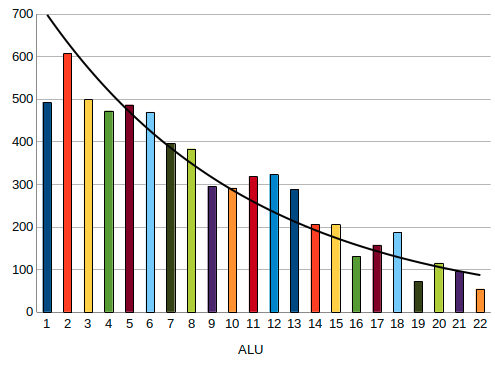

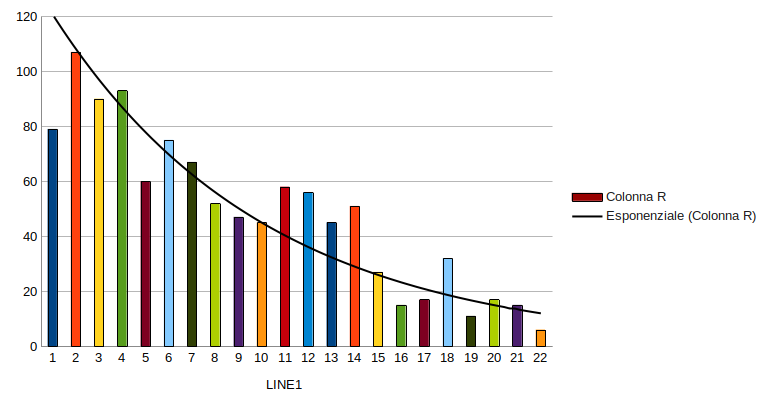

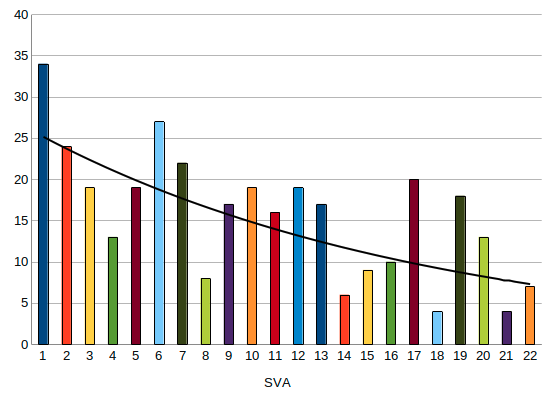


**Supplementary Figure 1.** A) Proportions of Alu, LINE-1 and SVA in the 22 autosomal chromosomes. B) Alu, LINE-1 and SVA content in the autosomal chromosomes.


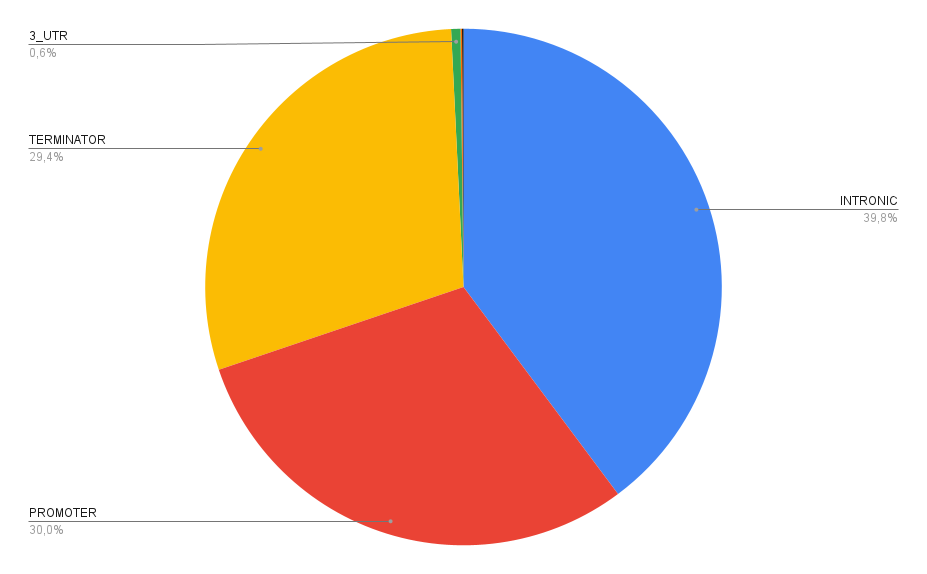


**Supplementary Figure 2.** Locations of the 7 952 nrTEs that are located into regulatory or genic regions: 39.8% are in introns (blue), 30.0% in promoters (red), 29.4% in terminators (yellow), 0.6% at the 3’ UTR (green), 0.1% at the 5’ UTR (orange) and 0.1% in exons (black).


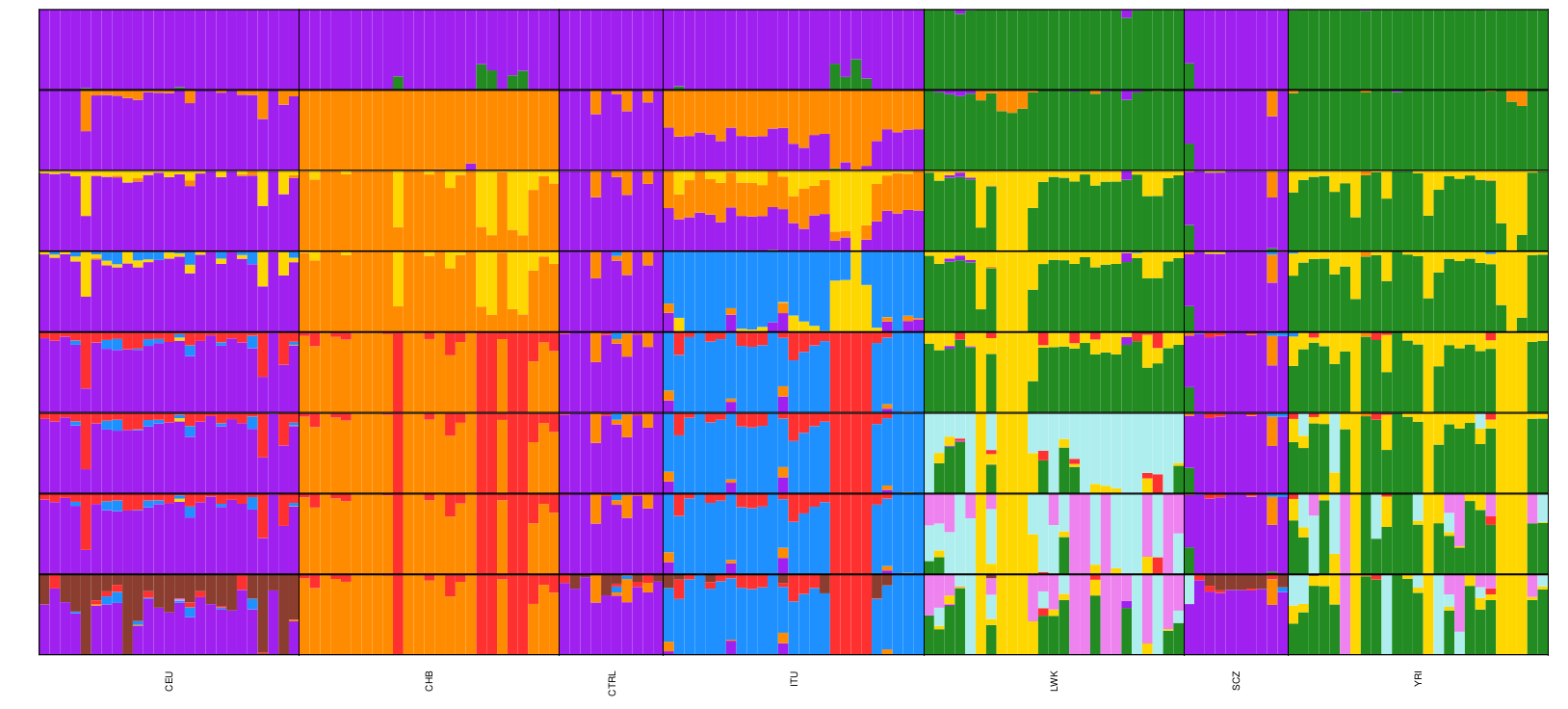


**Supplementary Figure 3.** Admixture plot of the 20 DLPFC samples and 125 1KGP individuals based only on nrTEs. A number K of putative ancestral components between 2 and 7 was tested (shown from top to bottom).


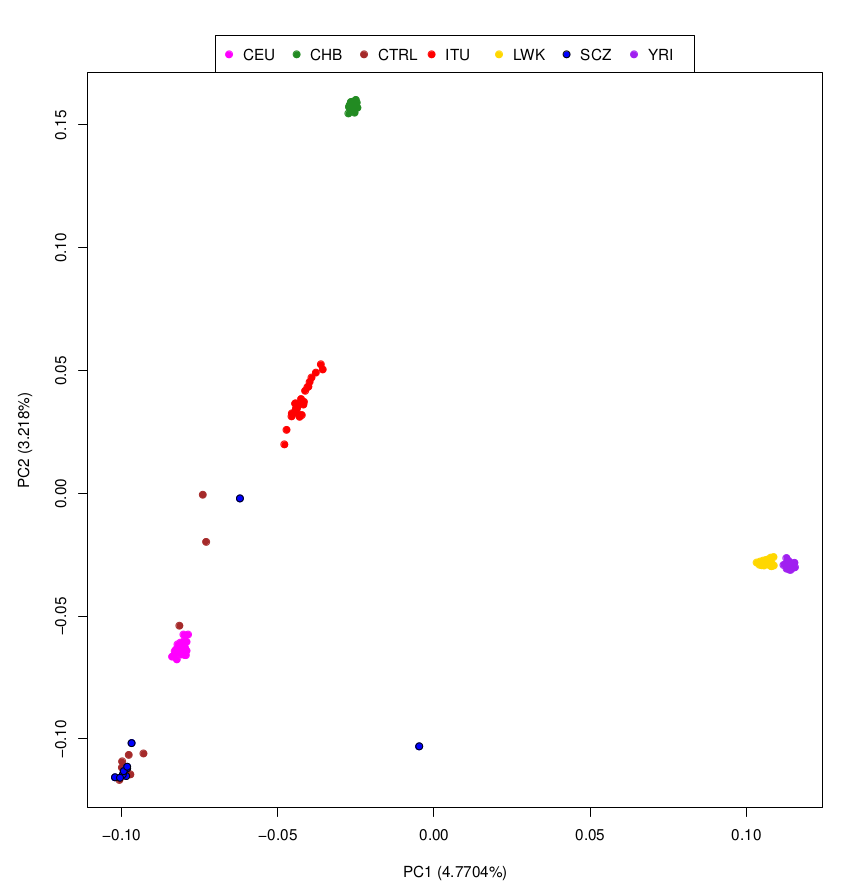


**Supplementary Figure 4.** Principal Component Analysis based on both SNPs and nrTEs. DLPFC samples cluster with Europeans, with the exception of two SCZ individuals who show signs of admixture with a sub-Saharan African source and an East Asian source, respectively. CEU=Europeans, CHB=Chinese, CTRL=controls, ITU=Indian Telugus, LWK=Luhya in Kenya, SCZ=schizophrenic individuals, YRI=Yoruba in Nigeria.


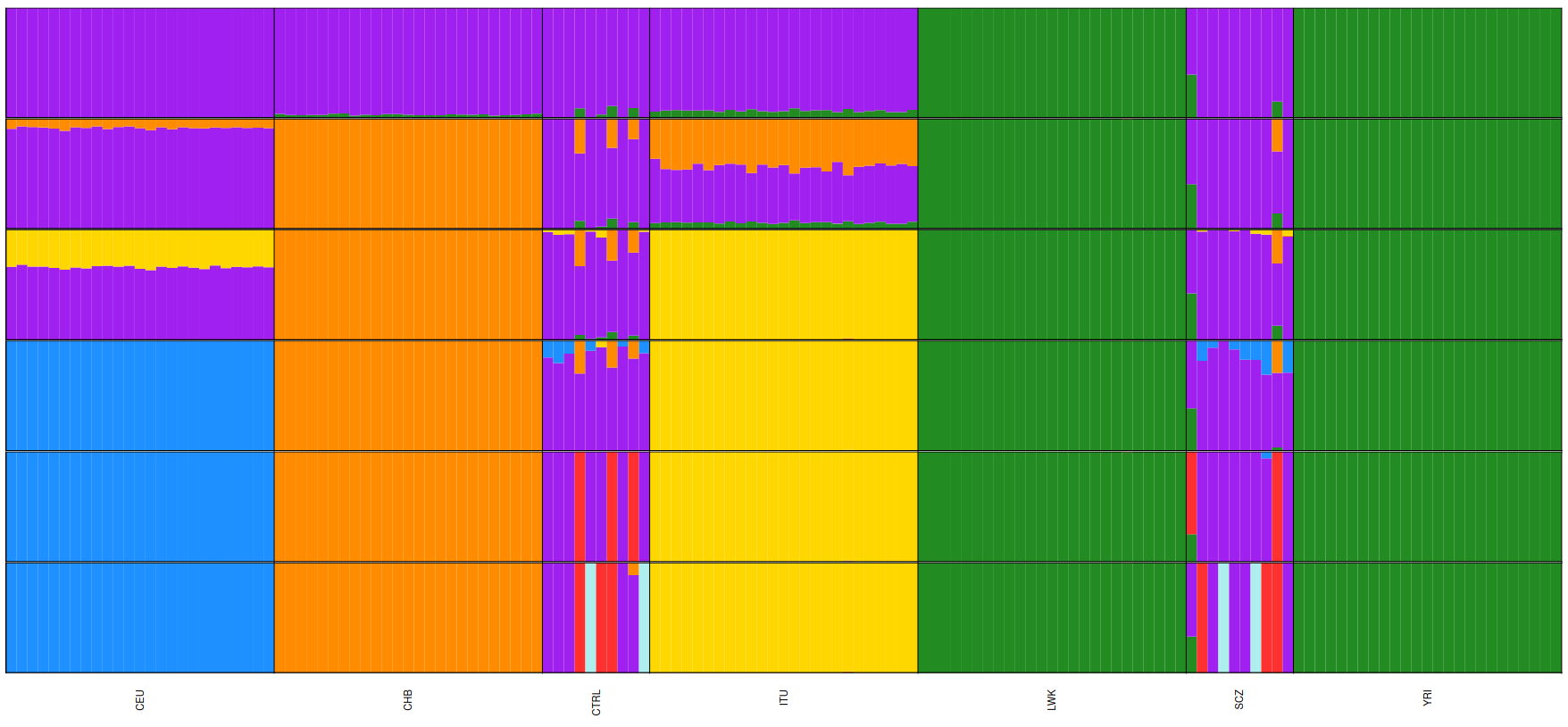


**Supplementary Figure 5.** Admixture plot based on the whole-variant dataset (nrTEs + SNPs).


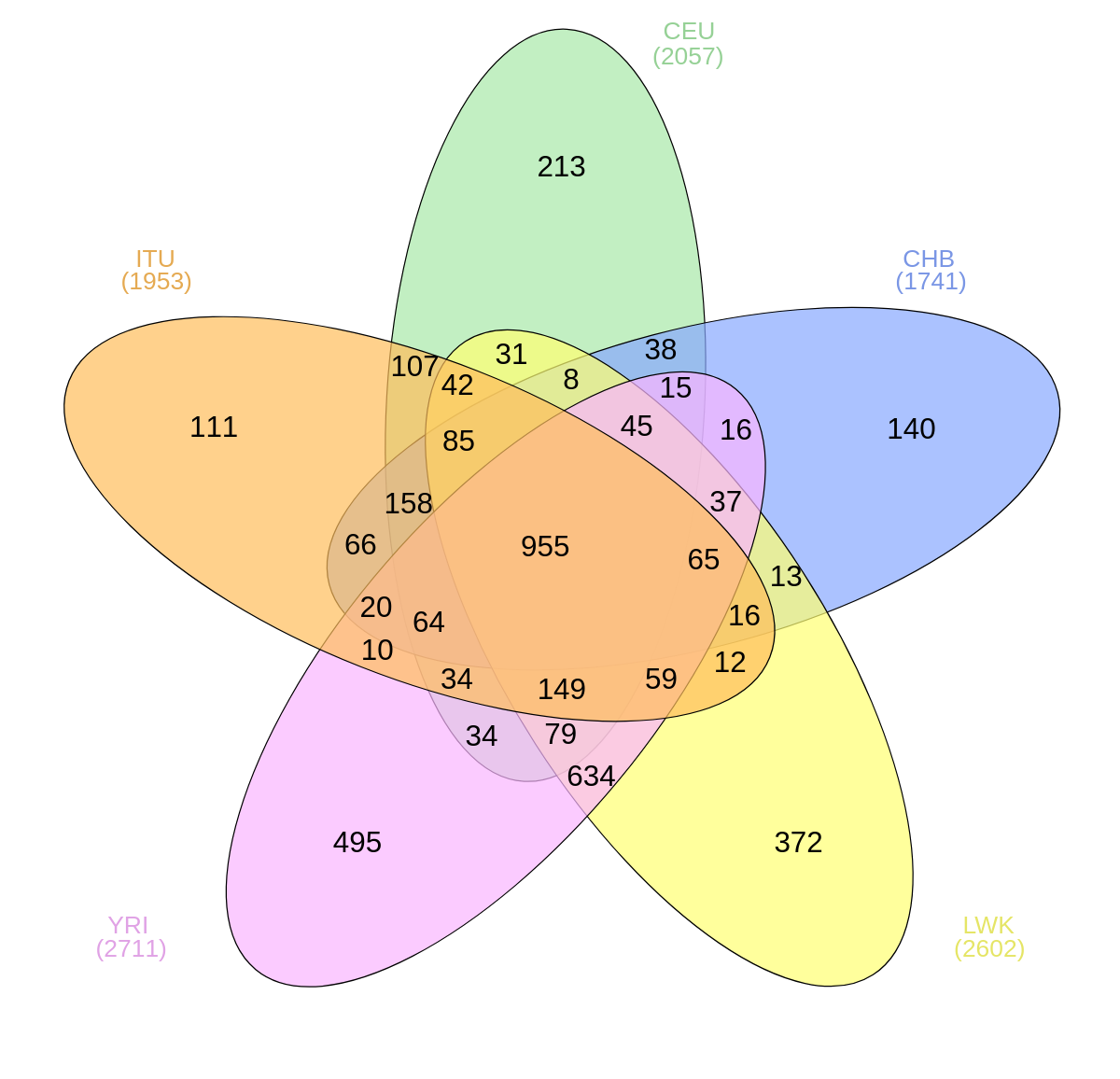


**Supplementary Figure 6.** Venn diagram showing the number of nrTEs with an allele frequency > 5% found across the five considered populations.


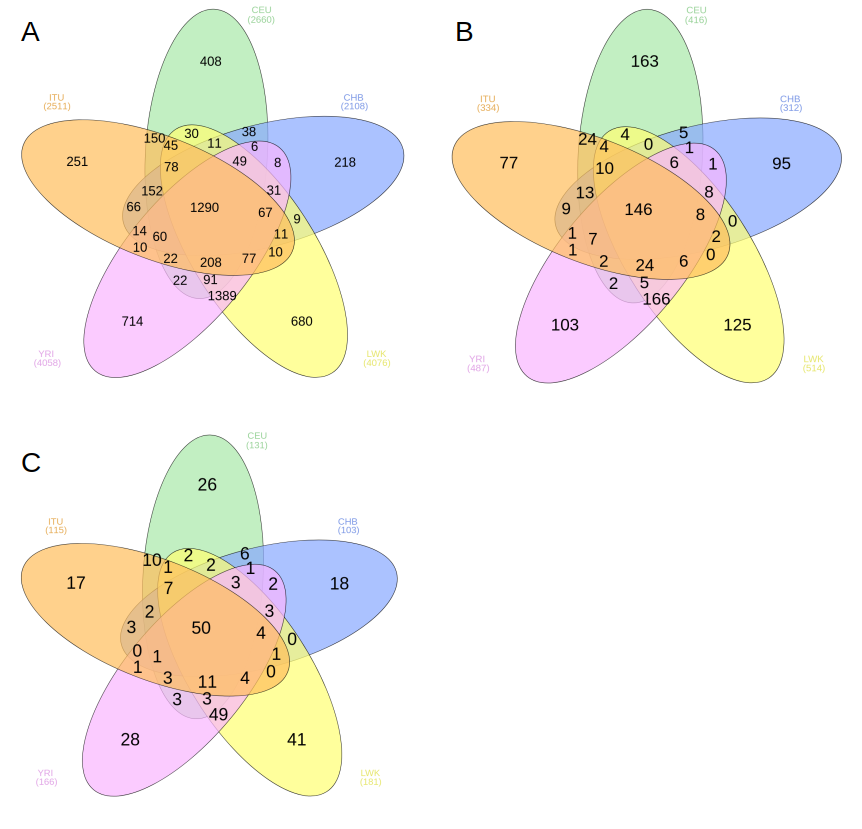


**Supplementary Figure 7.** nrTEs’ distribution in the five considered populations. A) Alu; B) LINE-1; C) SVA.
